# Transcriptome Analysis of Monozygotic Twin Brothers with Childhood Primary Myelofibrosis

**DOI:** 10.1016/j.gpb.2016.12.002

**Published:** 2017-02-07

**Authors:** Nan Ding, Zhaojun Zhang, Wenyu Yang, Lan Ren, Yingchi Zhang, Jingliao Zhang, Zhanqi Li, Peihong Zhang, Xiaofan Zhu, Xiaojuan Chen, Xiangdong Fang

**Affiliations:** 1 CAS Key Laboratory of Genome Sciences and Information, Beijing Institute of Genomics, Chinese Academy of Sciences, Beijing 100101, China; 2 College of Life Sciences, University of Chinese Academy of Sciences, Beijing 100049, China; 3 Sino-Danish College, University of Chinese Academy of Sciences, Beijing 100190, China; 4 State Key Laboratory of Experimental Hematology, Institute of Hematology and Blood Diseases Hospital, Chinese Academy of Medical Sciences & Peking Union Medical College, Tianjin 300020, China

**Keywords:** Primary myelofibrosis, RNA-seq, Apoptosis, Monozygotic twin, Childhood, Hematological disease

## Abstract

**Primary myelofibrosis** (PMF) is a chronic myeloproliferative disorder in human bone marrow. Over 50% of patients with myelofibrosis have mutations in *JAK2*, *MPL*, or *CALR*. However, these mutations are rarely detected in children, suggesting a difference in the pathogenesis of **childhood** PMF. In this study, we investigated the response to drug treatment of a **monozygotic twin** pair with typical childhood PMF. The twin exhibited different clinical outcomes despite following the same treatment regimen. The transcriptomic profiles of patient samples after drug treatment (E2 and Y2) were significantly different between the twin pair, which is consistent with the observation that the drug treatment was effective only in the younger brother, despite the twin being genetically identical. Bioinformatics analysis of the drug-responsive genes showed that the JAK-STAT pathway was activated in the cured younger brother, which is opposite to the pathway inhibition observed in adult PMF cases following treatment. Moreover, **apoptosis** and cell cycle processes were both significantly influenced by drug treatment in the sample of younger brother (Y2), implying their potential association with the pathogenesis of **childhood** PMF. Gene mutations in *JAK2*, *MPL*, or *CALR* were not observed; however, mutations in genes including *SRSF2* and *SF3B1* occurred in this twin pair with **childhood** PMF. Gene fusion events were extensively screened in the twin pair samples and the occurrence of *IGLV2-14-IGLL5* gene fusion was confirmed. The current study reported at transcriptomic level the different responses of **monozygotic twin** brothers with **childhood** PMF to the same androgen/prednisone treatment regimen providing new insights into the potential pathogenesis of **childhood** PMF for further research and clinical applications.

## Introduction

Primary myelofibrosis (PMF) is an agnogenic myeloid metaplasia resulting from abnormal clonal myeloid stem cells in the bone marrow (BM) [1], [2], [3]. PMF is characterized by BM diffuse fibrosis, splenomegaly, and extramedullary hematopoiesis, while the fibrous tissue produces few red blood cells (RBCs), leading to a failure of hematopoiesis [1], [2], [3]. PMF is frequently observed prior to the onset of other diseases such as acute myeloid leukemia (AML) [4], [5]. Effective approaches for the treatment of this disease include allogeneic hematopoietic stem cell transplantation (HSCT) [6] and splenectomy [7]. The incidence of PMF is approximately 1 case per 100,000 people, with a median age of onset of 60 years [8]. PMF is extremely rare in children [9], although sporadic childhood PMF cases have been described throughout the world [2], [6], [10]. Up till now, most studies have focused more on the clinical and cytogenetic phenotypes associated with the disease, rather than on drug therapies and related mechanisms.

*CALR*, *JAK2* and *MPL* encode calreticulin, Janus kinase 2, and myeloproliferative leukemia virus oncogene, respectively. Mutations in these genes are believed to directly or indirectly activate JAK-STAT pathway [1], [11] and have been reported to be important for the development of PMF in adults [1]. The *JAK2*-V617F mutation was reportedly present in approximately 50% of adult patients with PMF, and was associated with poor survival and an increased likelihood of transfusion dependence [12]. *MPL* mutations rarely coexist with the *JAK2*-V617F mutation in PMF patients [13], and have been associated with an increased risk of transfusion dependence and lower hemoglobin levels at diagnosis [14]. Tefferi et al. showed, in a study involving 254 patients, that *CALR* mutations were associated with young age, high platelet count, and low Dynamic International Prognostic Scoring (DIPSS)-plus score [15]. Although *CALR*, *JAK2*, and *MPL* have been described as causative genes in adult PMF, the causes of childhood PMF remain elusive. Among the three pediatric PMF cases reported by Slone and colleagues, only one case possessed a *JAK2*-V617F mutation and exhibited blindness, whereas the other two cases showed clinical courses that are rarely observed in adult PMF patients and thus remained agnogenic [16]. Therefore, the mechanisms underlying the pathogenesis of childhood PMF remain a mystery.

In this study, we examined a male monozygotic twin pair with PMF but showing discordant disease courses. Morphologically characterized as grade III PMF, these patients did not carry mutations in *JAK2*, *CALR*, or *MPL*. The patients underwent the same drug treatment, *i.e.*, androgen combined with prednisone; however, their responses were different. We analyzed the transcriptomes of the twin brothers before and after treatment, and identified drug-responsive genes. This allowed us to examine the underlying molecular mechanisms of differential responses to drug treatment and potential pathogenic mechanisms in childhood PMF.

## Results

### The identification of monozygotic twin brothers with childhood PMF

The monozygotic twin brothers were born in November, 2007. At one year of age they were diagnosed with recurrent epistaxis and skin ecchymosis, and then at two years of age they were diagnosed with an expanded abdomen. During this period, the patients developed hepatosplenomegaly with the liver and spleen measuring 1.5 cm and 3.0 cm below the costal margin, respectively in the elder brother, and 1.5 cm and 5.0 cm below the costal margin, respectively in the younger brother. Routine peripheral blood counts revealed anemia and thrombocytopenia in both patients. However, blood biochemistry, bleeding and coagulation behavior, and hemoglobin electrophoresis tests appeared to be normal (Table S1). The initial treatments administered at local hospitals were unknown, which did not relieve the symptoms of the patients. The patients underwent discontinuous blood infusion treatment before being admitted to the Blood Disease Hospital at the Chinese Academy of Medical Sciences (CAMS), Peking Union Medical College (PUMC) at four years and seven months of age.

### Cytogenetic and morphological analysis and therapy choices

**Figure 1:**
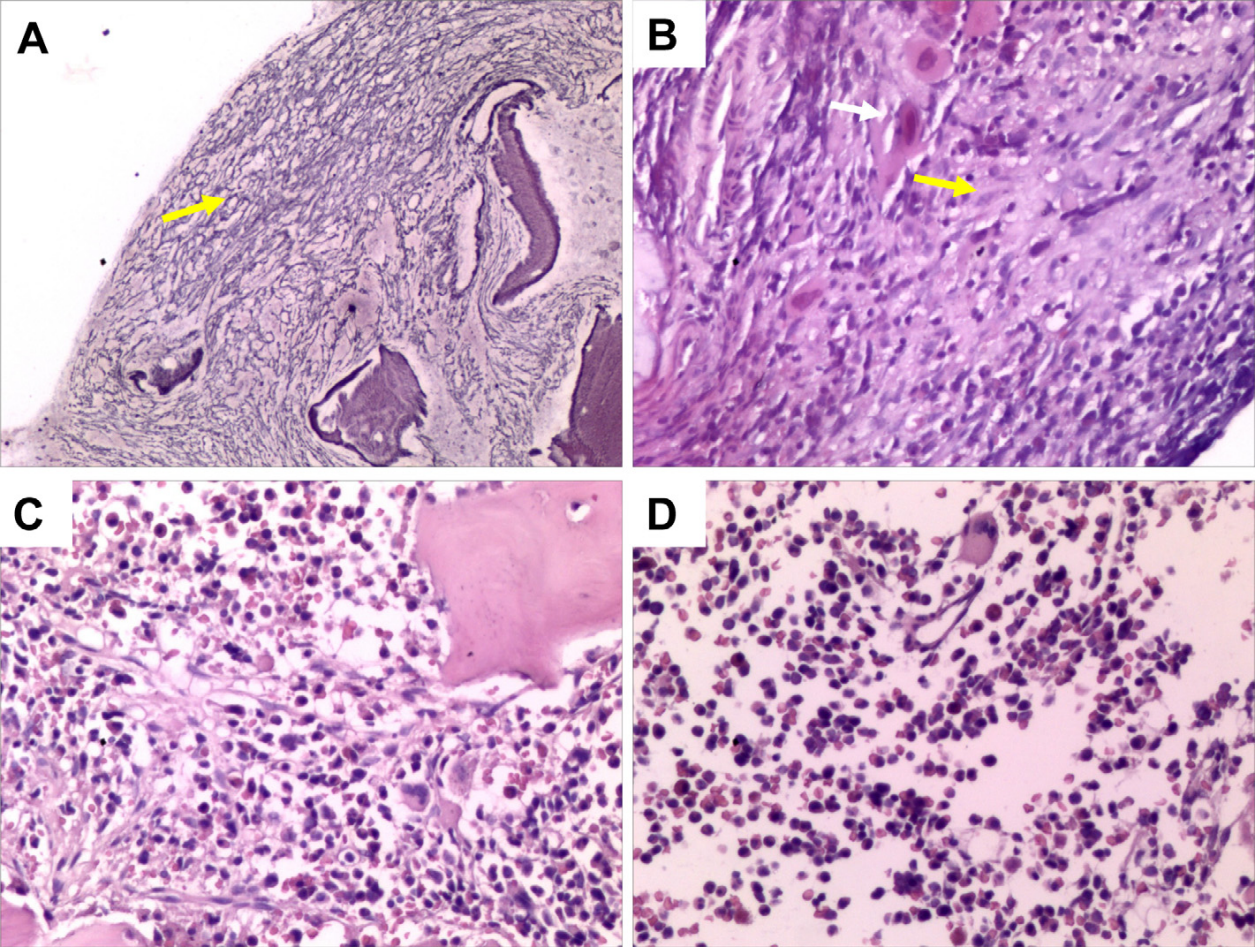
**Histological analysis of bone marrow biopsies from monozygotic twin brothers before and after treatment** **A**. Grade III fibrosis in sample E1 (silver staining, 100× magnification). **B**. Substantial collagen fibrosis with an associated stream-like arrangement of megakaryocytes in sample E1. White arrow indicates megakaryocyte, and yellow arrow indicates collagen fibrosis (H&E staining, 200× magnification). **C**. Partial collagen fibrosis and megakaryocytes showing a mildly atypical morphology in sample E2 (H&E, 200× magnification). **D**. Mildly atypical megakaryocytes with no collagen fibrosis in sample Y2 (H&E, 200× magnification).

Smear examination of the patients’ BM samples prior to drug treatment showed hyperactive hyperplasia of mature granulocytes and RBCs, as well as megakaryophthisis (Table S1). Additionally, a slight increase in immature cells was also observed in the patients’ BM biopsies. Megakaryocytes with abnormal morphology were detected in the BM, and there was extensive fibrous tissue hyperplasia that was classified as grade III reticulin fibers, supporting the diagnosis of primary myelofibrosis (Figure 1A and B; Table S1). PCR amplification and sequencing analysis showed that no mutations in *JAK2*, *MPL*, and *CALR* were detected (data not shown).

Based on these symptoms, both patients underwent drug treatment with oral androgen (2 mg/d) and prednisone (5 mg/d). Surprisingly, the twin brothers responded differentially to the same treatment. In the younger brother, the enlargement of the liver and spleen was significantly attenuated, and he became transfusion independent. At a reexamination at five years and five months of age, his liver and spleen had both decreased to normal size. Additionally, his BM biopsy was normal as well. The younger brother continued to take androgen, but not prednisone. At a reexamination when he was seven years old, he was diagnosed as cured and discontinued treatment completely.

**Figure 2:**
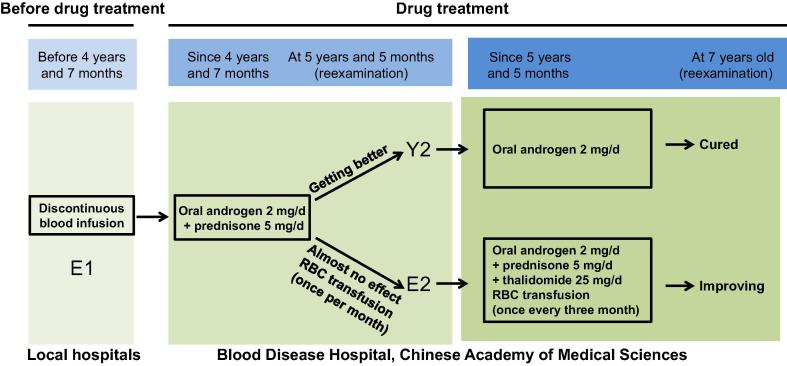
**Clinical treatments for monozygotic twin brothers** The boxed areas describe treatment regimens for each of the patients. E1 and E2 denote bone marrow samples taken from the elder brother, before and after treatment, respectively; Y2 denotes the bone marrow sample taken from the younger brother after treatment. RBC, red blood cell.

The elder brother, however, continued to display hepatosplenomegaly, with the liver and spleen measuring 2 cm and 15 cm below the costal margin, respectively, at 5 years and 5 months of age. A discontinuous RBC transfusion treatment was given to this patient once a month, and platelet infusion was discontinued between four years and seven months of age and 5 years and 5 months of age. At five years and five months of age, collagen fibrosis with atypical morphology was readily detected (Figure1C). In addition, megakaryophthisis, active hyperplasia of granulocytes and RBCs, and pancytopenia revealed by blood counts continued to be evident. Clearly, the drug treatment was less effective in the elder brother than in the younger brother (Figure 1C and D; Table S1). Therefore, treatment regimen for the elder brother was adjusted at five years and five months of his age. This includes adding oral thalidomide (25 mg/d) to androgen/prednisone and lowering the frequency of RBC transfusion to once every three months on average. Following these adjustments, the elder brother was improving at the reexamination when he was seven years of age (Figure 2). He had normal white blood cell and platelet counts and physical examination showed spleen measuring 3 cm below the costal margin without hepatomegaly.

### Transcriptome analysis of monozygotic twin brothers with PMF

**Figure 3:**
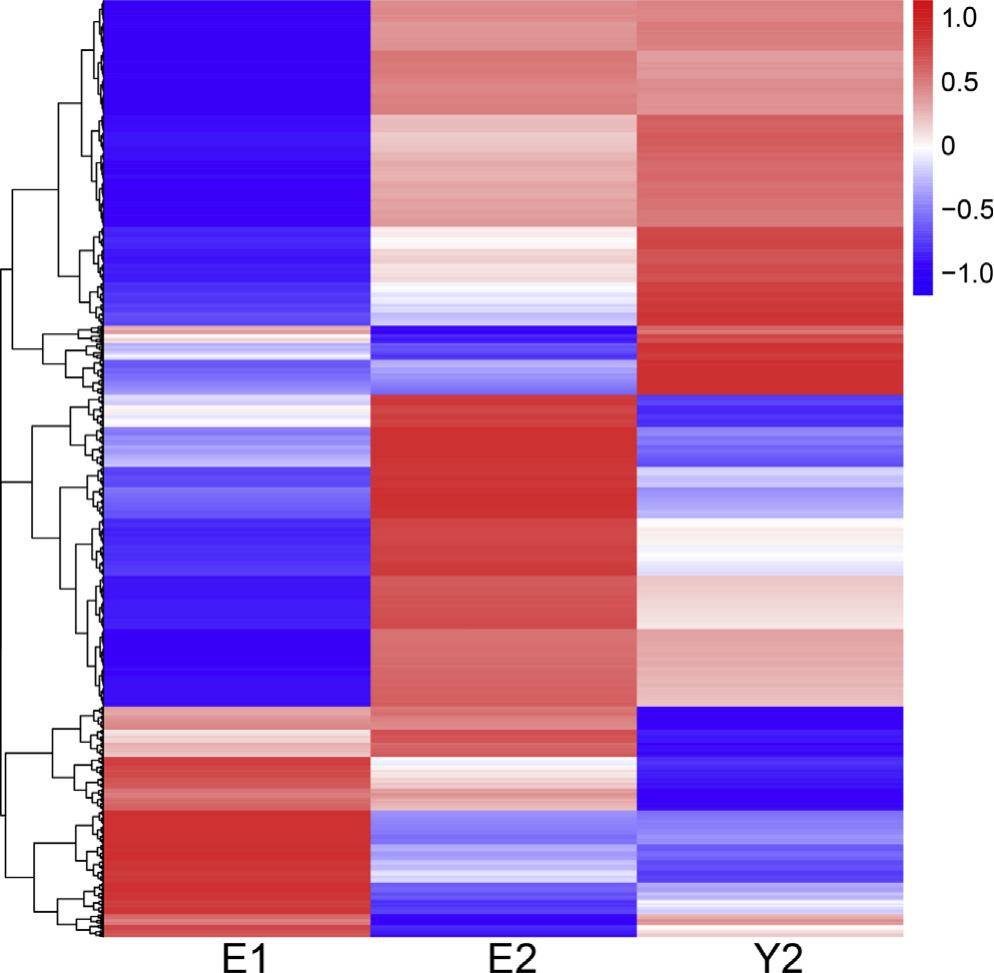
**A heatmap of expressed genes in the twin brothers** A large number of genes were lowly expressed in the elder brother before he received treatment, but showed up-regulated expression after the patients received treatment. The expression profiles for the elder brother before and after treatment are labeled by E1 and E2, respectively, while Y2 denotes the expression profile of the younger brother after treatment. Red and blue bars represent genes with high or low expression levels, respectively. The heatmap was generated using unsupervised hierarchical clustering, and the Pearson correlation coefficient was calculated as described in the “Materials and methods” section.

To investigate the possible mechanisms underlying the differential responses of the twin pair to identical drug treatment regimen, the transcriptomes from the BM samples before and after treatment were analyzed using an Illumina sequencing platform. We obtained 16,299,013 and 14,148,939 sequencing reads for the elder brother before (E1) and after (E2) drug treatment, respectively, of which 76.5% and 76.9% were mapped to the reference human genome. The number of sequencing reads for the younger brother after treatment (Y2) was 20,669,243, of which 77% were mapped to the reference human genome. The BM sample from the younger brother before treatment (Y1) was not collected for sequencing purpose. In total 11,910 expressed genes were identified with a cutoff of fragments per kilobase of exon per million (FPKM) >1 in at least one sample. The transcriptome profiles of the monozygotic twin brothers differed following treatment (Figure 3), which is consistent with the differential drug responses of the twin despite being genetically identical. The younger brother showed clear progress after 10 months of treatment, but the androgen/prednisone drug combination was clearly ineffective in the elder brother. The expression of 1451 genes was significantly altered in the elder brother following treatment, although we could not rule out the possibility that these could be attributed to drug-induced non-specific perturbations. On the other hand, some genes were expressed at the comparable levels in the elder brother before and after treatment, whereas significant alterations in the expression of these genes were revealed in the younger brother following treatment. In total, 1187 genes were differentially expressed between E1 and Y2 (fold change >2, *P* < 0.05). Among them, expression of 824 genes remained similar without showing significant changes between E1 and E2 (Table S2). These genes were defined to be responsive to the androgen/prednisone drug treatment regimen, which could be potential causative genes for childhood PMF.

### Gene set enrichment analysis of genes responsive to drug treatment

**Figure 4:**
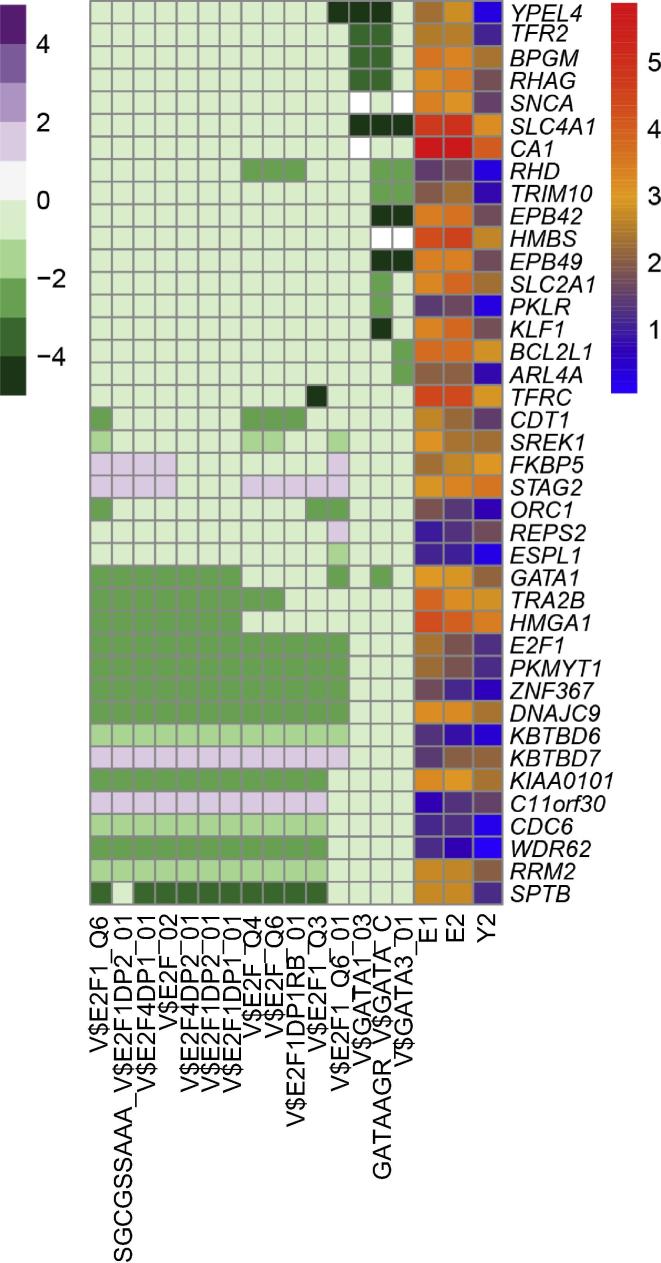
**Profiling of gene expression and transcription factor enrichment for 40 DEGs regulated by E2F and GATA** Drug-responsive genes were subjected to gene set enrichment pre-ranked analysis. The purple-white-green heatmap shows the enrichment of transcription factors potentially regulating the DEGs, and the red–orange–blue heatmap shows the expression defined as FPKM for each gene. Genes were ranked according to the fold change in expression between E1 and Y2 samples. FPKM, fragments per kilobase of exon per million; DEG, differentially-expressed gene.

We performed the gene set enrichment analysis (GSEA) on the 824 drug-responsive genes to find regions of chromosomal enrichment and potential transcriptional regulators of these genes. Totally 49 drug-responsive genes were clustered at the chr19p13 and chr19q13 regions. Of these, expression of 34 genes was up-regulated after treatment, whereas expression of the remaining 15 genes was down-regulated in Y2 (Table 1). Chromosome 19 is therefore likely to be the genomic region that is most sensitive to drug treatment in this case. We also performed transcription factor (TF) enrichment analysis on these 824 drug-responsive genes to identify TFs that potentially regulate their expression. As a result, 40 genes were found to be potentially regulated by the TFs E2F and GATA (Figure 4).

### Drug treatment affects apoptosis and cell cycle

**Figure 5:**
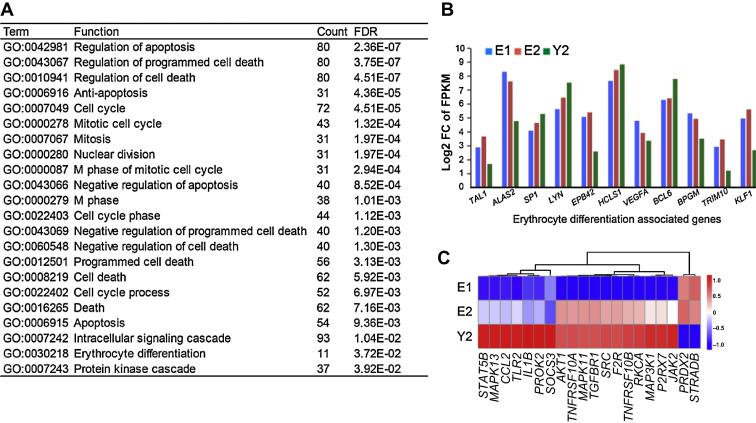
**Enriched functions for genes that are differentially expressed between the twin brothers after treatment** **A.** GO analysis was performed using the DAVID tool, with a FDR <0.05 defined as significant. **B.** Bar plot shows the expression (FPKM) of 11 genes associated with erythrocyte differentiation across the three samples. **C.** The expression profiles of 20 genes that are frequently associated with the regulation of apoptosis, erythrocyte differentiation, and cell cycle. Red and blue bars represent high and low levels of expression, respectively. E1 and E2 denote the BM samples from elder brother before and after treatment, respectively, and Y2 denotes the BM sample from younger brother after treatment. FPKM, fragments per kilobase of exon per million; FDR, false discovery rate; FC, fold change.

Gene Ontology (GO) analysis was performed to identify functions that are enriched in the drug-responsive gene set. Biological processes associated with apoptosis, the cell cycle, and erythroid differentiation were found to be enriched (Figure 5A). Alterations in expression of genes that are associated with apoptosis and cell cycle suggest that drug treatment could significantly affect these biological processes in patients with childhood PMF. PMF is often accompanied by erythrocyte disorders in patient [17]. Notably, our transcriptomic analysis showed that factors associated with erythroid differentiation, such as *TAL1*, *ALAS2*, *SP1*, *LYN*, *EPB42*, *HCLS1*, *VEGFA*, *BCL6*, *BPGM*, *TRIM10*, and *KLF1*, were differentially expressed in both E2 and Y2 samples (Figure 5B). The enrichment for erythroid differentiation factors is probably related to abnormal extramedullary hematopoiesis observed in PMF [17]. This generally can be treated with low doses of thalidomide together with prednisone to modulate the activity of the immune system and prevent angiogenesis, thus preventing myelofibrosis and improving hematopoiesis in the BM [18].

### The JAK-STAT pathway is activated in the younger brother after drug treatment

**Figure 6:**
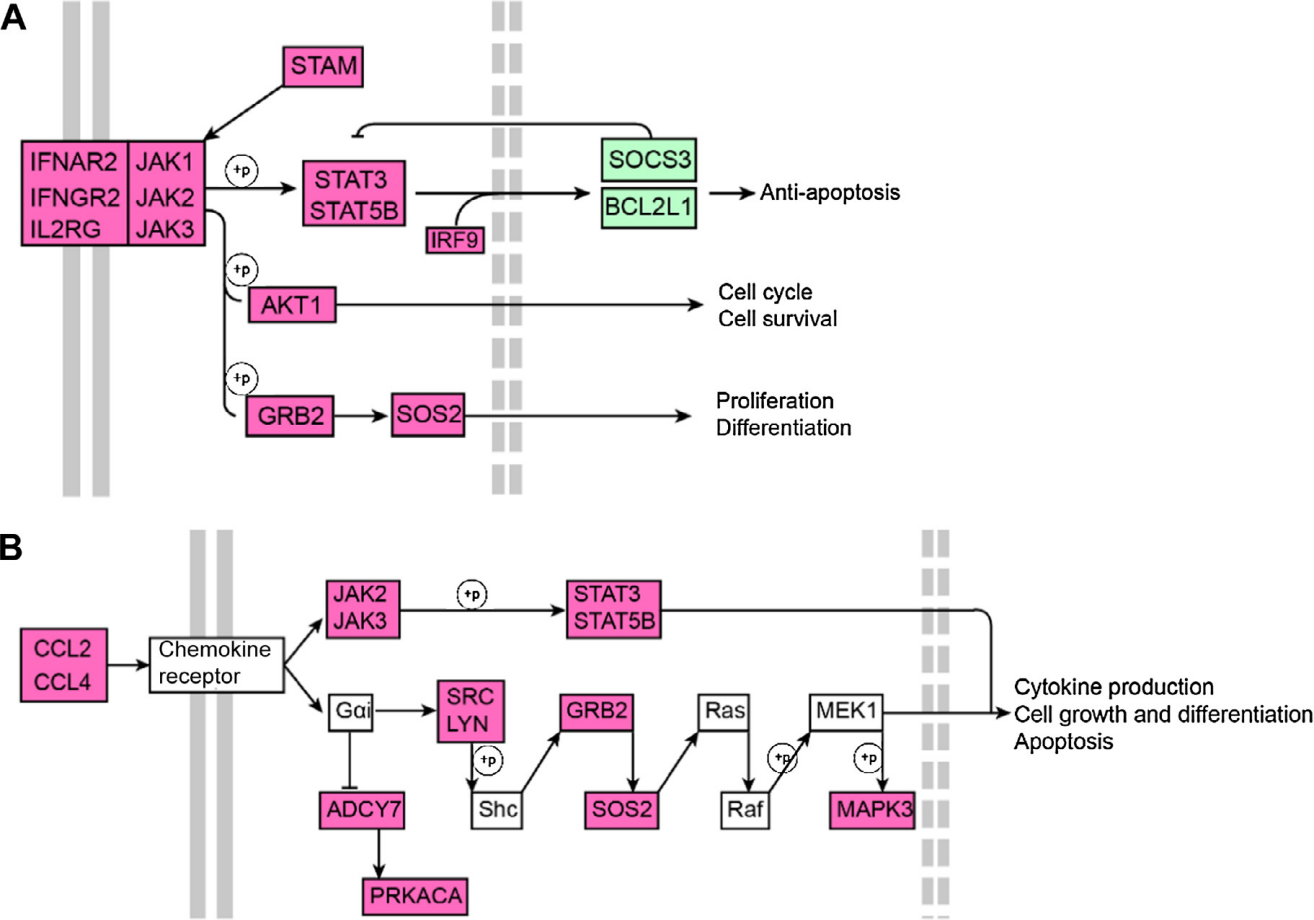
**JAK-STAT and chemokine signaling pathways** **A.** The JAK-STAT signaling pathway was activated in the Y2 sample. **B.** The chemokine signaling pathway was also activated in the Y2 sample. Expression of JAK-family coding genes was up-regulated in the Y2 sample. Red and green nodes indicate coding genes with up-regulated and down-regulated expression in the Y2 sample, respectively.

While investigating the key genes involved in childhood PMF, we found that 20 genes, including *JAK2*, were involved in all of the GO functions enriched in the drug-responsive set (Figure 5C). These genes, including *AKT1*, *JAK2*, *BCL2L1*, *SOS2*, *STAT5B*, and *SOCS3*, are associated with the activation of the JAK-STAT signaling pathway (Figure 6). JAK-STAT signaling pathway has been shown to be inhibited in patients that are cured of adult PMF [1]. Therefore, molecular response to combined androgen/prednisone drug treatment seems to differ markedly between patients with childhood PMF and those with adult PMF. The V617F mutation of *JAK2* is well-studied as the main cause for myelofibrosis [7]; however, this mutation was not detected in the twin pair patients examined in this study. Instead, we detected another two synonymous SNPs in the coding sequence of *JAK2*, suggesting the existence of undiscovered causative mechanisms in the development of childhood PMF.

### Fusion genes are detected in childhood PMF

**Figure 7:**
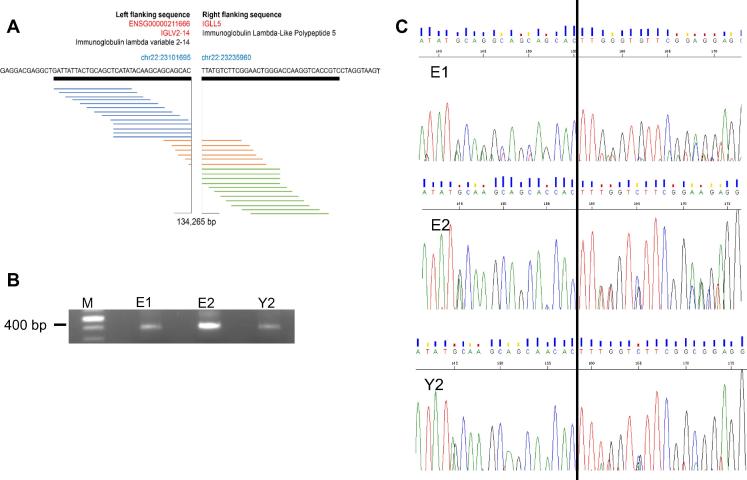
**The fusion gene *IGLV2-14-IGLL5* detected in monozygotic twin brothers with PMF** **A.** A representative fusion gene of *IGLV2-14-IGLL5* on chromosome 22 was detected from transcriptomic sequencing data. **B.** The *IGLV2-14-IGLL5* fusion gene was confirmed by PCR amplification. M represents marker. The expected size of 378 bp is marked with an arrow. **C.** The Sanger sequencing profiles for three samples. The break points were marked with a vertical line. E1 and E2 denote the elder brother before and after treatment, respectively, and Y2 denotes the younger brother after treatment.

The significant changes in gene expression between the E2 and Y2 prompted us to further investigate the pathogenesis of PMF at the whole transcriptome level. The investigation of nonrandom chromosomal translocation breakpoints has led to the identification of many genes crucial for malignant transformation and shed light on the pathogenesis and prognosis of hematopoietic diseases [19]. We therefore performed fusion detection analysis at the transcriptome level to find potential fusion genes. Paired-end reads were mapped to the reference human genome, in which the two ends mapped to different genes or reads spanning two genes were considered indicative of potential fusion candidates. Consequently, a total of 23 gene fusions were identified in twin brothers by using TopHat-fuse analysis (Table 2). The *IGLV2-14-IGLL5* gene fusion had the highest number of reads and was selected for scrutinization. *IGLV2-14* and *IGLL5* are both located on chromosome 22 but they are 134,265 bp away from each other. The *IGLV2-14-IGLL5* gene fusion occurred between exon 2 of *IGLV2-14* and exon 2 of *IGLL5*, and was identified in patients for the first time in the current study. *IGLV2-14* encodes immunoglobulin lambda variable 2–14, which is likely to be associated with chronic lymphocytic leukemia (CLL) [20]. *IGLL5* encodes the immunoglobulin lambda-like polypeptide 5, which has not been extensively investigated yet. *IGLL5* was expressed in lymphomas [21]. Is of note that in lymph nodes breast cancer patients, *IGLL5* is frequently fused with variants of *IGLV1* that is located ∼ 500 kb upstream of *IGLL5* [22]. Our sequencing and PCR analyses validated the presence of this fusion gene in twin brothers before and after drug treatment (Figure 7).

To confirm whether the fusion gene is present in other childhood hematological diseases, we also examined BM samples of childhood Diamond-Blackfan anemia (DBA) and idiopathic thrombocytopenic purpura (ITP). The fusion gene was detected in DBA, but not in childhood ITP patient or normal human umbilical cord blood mononuclear cells (UBMCs) (data not shown), indicating the fusion gene of *IGLV2-14-IGLL5* is not specific for childhood PMF. Taken together, these findings show that fusion genes could be the potential causative agents in the pathogenesis of childhood PMF.

### *SRSF2* and *SF3B1* gene mutations occurred in both adult and childhood PMF

Besides *JAK2*, *MPL*, and *CARL*, mutations in other genes, including *ASXL1*, *EZH2*, *IDH2*, and *U2AF1*, are known to have prognostic relevance in PMF as well [23], [24]. In this study, we comprehensively screened samples from the patients with childhood PMF for gene mutations (Table S3). An average of 5754 SNPs (5455 in E1sample, 5694 in E2 sample, and 6114 in Y2 sample) were identified using the RNA-seq data. This screen confirmed that no mutations occurred in the *JAK2*, *CALR*, or *MPL* genes in any of the samples. To identify gene mutations relevant to childhood PMF, we focused on the 4122 SNPs that were shared among the three samples. Two genes with mutations were found to be differentially expressed in the younger brother, including *SRSF2* and *SF3B1*. Mutations in these two genes have been shown to be relevant to adult PMF by influencing RNA splicing [1]. There are more than one missense mutation found in *SRSF2* and *SF3B1* in each of the three samples (SNPs rs237057 and rs237058 for *SRSF2*; rs788018 and rs788023 for *SF3B1*), respectively. All these four mutations have also been detected in pediatric patients with AML [25]. In summary, although no mutations were detected in the genes *JAK2*, *MPL*, and *CALR*, two gene mutations involved in both the adult and childhood forms of PMF were detected. The pathogenic role of such mutations in disease progression in both adult and childhood PMF remains poorly understood.

## Discussion

PMF is a severe form of myeloproliferative neoplasms, and fibrosis of the BM causes abnormal hematopoiesis both in the BM and in other organs. It is rare in children, and only a handful of childhood PMF cases, accompanied by other diseases and with disparate clinical symptoms, have been reported to date [6], [10]. A close relationship between PMF and leukemia has been demonstrated by Tavares, with PMF diagnosed either prior to acute leukemia or after leukemia treatment (http://emedicine.medscape.com/article/956806-overview#a3). In the current study, we noted that prior to androgen/prednisone drug treatment, the monozygotic twin brothers exhibited typical reticulin fibers (grade III) in BM, as well as palpable hepatosplenomegaly, anemia, megakaryophthisis, and active hyperplasia of granulocytes and RBCs. The absence of mutations in the known causative genes *JAK2*, *CALR*, and *MPL* suggests that the pathogenic mechanisms in the current pediatric PMF case could be attributed to other genetic variations.

It is of note that the twin brothers, despite with identical genetic backgrounds, exhibited different responses to the same drug treatment regime. The point mutations and gene fusions detected in the current study occurred in both brothers, and did not differ before and after drug treatment. We thus speculate that the difference in response to treatment could be due to specific changes at the epigenetic or transcriptional level. Interestingly, we observed alterations in the expression of genes relevant to epigenetic regulation, including *TET2* and *DNMT3A*, which have previously been reported to be associated with PMF (Table S2) [1]. *JAK2* is responsible for the activation of molecules involved in RBC production [26], and thus the increase in *JAK2* gene expression in the cured younger brother is probably involved in the disappearance of his anemia symptoms. Therefore, transcriptional changes leading to enhanced expression of the genes of the JAK/STAT pathway could, in concert with epigenetic changes, explain the different responses to treatment.

The genetic profiles of adult patients with PMF have been extensively studied, and a number of promising gene candidates have been reported. However, very few studies have been performed on pediatric patients with PMF, which could be partially due to the rarity of pediatric PMF. In this study, we have investigated the mechanisms underlying the response to treatment with androgen and prednisone, as well as potential causative factors in childhood PMF, by comparing the transcriptome profiles of identical twin brothers before and after treatment. We reported that disturbances in apoptotic and cell cycle processes are likely to have roles in the development of childhood PMF. The JAK2-STAT pathway is known to be hyper-activated in adult PMF as a result of the mutation *JAK2*-V617F [1], and is inhibited by drug treatments received by these patients. Surprisingly, the JAK2-STAT pathway was found to be activated in the twin brothers following treatment, demonstrating that while childhood PMF involves different pathogenic mechanisms, the same pathway is involved. We found that the expression profiles of the twin brothers differed after 10 months of drug treatment. As the patients displayed discordant outcomes following the same treatment regimen, we examined those genes that were differentially expressed between the patients. These genes were defined as drug-responsive, supporting our hypothesis that differential gene expression is important in the response to drugs. Such differential gene expression could be the possible cause of the poor response to treatment of the elder brother.

Interestingly, a total of 49 differentially-expressed genes (DEGs) were clustered at the chromosomal locations chr19p13 and chr19q13. Forty of these genes possess binding motifs for the GATA and E2F TF families, suggesting that regulation by these factors, or proteins with similar DNA-binding domains, could be responsible for the differential response to treatment observed in these patients. The *BCR-ABL* fusion gene, caused by the reciprocal translocation of chromosomes 9 and 22, is a well-documented causative agent in chronic myeloid leukemia (CML) [27]. However, the PMF patients in this study were shown to be *BCR-ABL*-negative (data not shown). Due to limitations in the genomic data, we were unable to verify the existence of genomic mutations or instability at chromosome 19 in our patients with childhood PMF. The interesting observation that a number of DEGs were clustered in two specific regions of chromosome 19 would be investigated further.

Our GO analysis showed that the drug-responsive genes were associated with apoptosis, the cell cycle, and erythroid differentiation. To identify a key gene with multiple cellular roles, we examined 20 genes that were frequently associated with the enriched functions. Most of these genes are either regulated by *JAK2* or inhibit *JAK2* [28], [29], [30], [31], [32], [33]. In adults with myelofibrosis, which is commonly associated with the *JAK2-*V617F mutant, symptoms were relieved by the JAK inhibitor ruxolitinib [31]. Verstovsek et al. evaluated the long-term outcomes of 107 adult myelofibrosis patients who received ruxolitinib and found that the ruxolitinib treatment was associated with better overall survival [32]. However, ruxolitinib would be ineffective in the current case, as the JAK-STAT pathway was inactive in the uncured elder brother. The expression of *JAK2*, one of the 20 genes considered, was significantly higher in the BM of the cured patient than in the uncured patient. The higher expression of the *JAK1*, *JAK2*, and *JAK3* genes and their downstream targets *AKT1*, *STAM*, and *GRB2* in the cured patient indicates that the JAK-STAT signaling pathway was activated in the BM, possibly to regulate the cell cycle and apoptotic processes. Cytokines rely on the *JAK* family for control of cell growth and immune responses (Figure 6) [21]. We hypothesize that the drug-induced activation of *JAK2* gene expression enhances the apoptotic response to DNA damage. Taken together, our results suggest the involvement of different pathogenic mechanisms in childhood PMF, and imply that the *JAK* gene family is involved in this disease in a manner that is independent of the documented *JAK2*-V617F mutation.

## Conclusion

This study, examining a monozygotic twin pair with childhood PMF, provides a suitable model for the investigation of the pathogenesis of PMF as well as the underlying molecular mechanisms involved in the response to drug treatment. We find that following treatment, the brothers have very different clinical outcomes and transcriptome profiles. In the cured PMF patient, the response to treatment is found to involve apoptosis, the cell cycle, and erythrocyte differentiation. While the JAK-STAT pathway is activated in the cured pediatric patient, it has previously been shown to be inhibited in cases of adult PMF. Gene fusion and mutations are found to occur in twin brothers with childhood PMF. In summary, this study provides valuable clinical and sequencing information for a monozygotic twin pair with rarely-seen childhood PMF, which would aid in a better understanding of childhood PMF.

## Materials and methods

### Sample preparation

A pair of four-year-old twin brothers, whose monozygosity was confirmed by hospital records, was diagnosed with PMF on the basis of clinical examination, blood counts and morphology. After written informed consent was obtained from the patients’ guardians, one BM sample was collected from the elder brother before treatment (at 4 years and 7 months of age) and each BM sample from both twin brothers after treatment (5 years and 5 months of age) for transcriptome sequencing. The twin brothers showed almost exactly the same clinical features and morphologies before treatment, and at this point a BM sample was collected from the elder brother only. Sample collection was performed with the permission of the children’s parents, and was approved by the Ethics Committee of the Institute of Hematology, Blood Disease Hospital, CAMS, PUMC.

### RNA preparation, library preparation, and sequencing

Total RNA was extracted using TRIzol Reagent (Invitrogen, Carlsbad, CA) according to the manufacturer’s instructions. RNA integrity was measured using 1.2% agarose gel electrophoresis, and residual genomic DNA was removed with RNase-free DNase I (Ambion, Austin, TX). mRNA libraries were constructed using an Illumina mRNA-Seq Library Preparation Kit. The size distribution and concentration of the mRNA in the libraries were determined using an Agilent Bioanalyzer DNA 2000 Chip (Agilent Technologies, Santa Clara, CA). The RNA-seq libraries were sequenced on an Illumina HiSeq2000 platform (Illumina, San Diego, CA), using a 101-bp, paired-end, non-strand-specific sequencing strategy.

### Data processing

Quality control analysis of the raw data was performed using FASTQC software (http://www.bioinformatics.babraham.ac.uk/projects/fastqc). The human hg19 reference genome was downloaded from the iGenomes database, and the associated .GTF files were downloaded from the Ensembl website (http://asia.ensembl.org/index.html). Raw sequencing reads (.fastq format) were mapped to the hg19 reference genome using TopHat version 2.0.9 software with default parameters [33], [34]. The Cuffdiff tool was used to identify DEGs using default parameters [35], [36]. Transcript abundance was defined as FPKM for RNA-seq data. A gene is considered expressed with FPKM >1 in at least one sample and significantly differentially-expressed with fold change >2 and *P* < 0.05. Genes that were differentially expressed before and after treatment were grouped for clustering analysis. K-means clustering was performed using the R package ConsensusClusterPlus [37]. Enrichment analysis for GO biological processes and TFs, as well as hallmark analysis, was performed by using GSEA [38]. Tophat-fusion-post was used to filter out false fusions and annotate fusion genes [39]. Global gene fusions were further filtered to include those with ≥10 reads spanning the break-point [39]. SNPs were called using GATK, SAMtools, and BEDtools according to the pipeline described in SNPiR [40]. The transcriptome data had been deposited in the Genome Sequencing Archive (accession No. PRJCA000343).

### Unsupervised clustering

Unsupervised hierarchical clustering of filtered gene expression profiles (FPKM >1) was performed using R software to examine relationships between the three samples [41]. The distance (*d_XY_*) between genes and samples was evaluated using the Pearson correlation coefficient (*r_XY_*), where *d_XY_* = 1 − *r_XY_*.

The Pearson correlation coefficient function is defined as:

$$r=\frac{1}{n-1}\overset{n}{\underset{i=1}{\sum }}\left(\frac{X-\overset{‾}{X}}{S_{X}}\right)\left(\frac{Y-\overset{‾}{Y}}{S_{Y}}\right)$$

where

$$S_{X}=\frac{1}{n-1}\sqrt{\overset{n}{\underset{i=1}{\sum }}{\left(X_{i}-\overset{‾}{X}\right)}^{2}}\text{,}\;S_{Y}=\frac{1}{n-1}\sqrt{\overset{n}{\underset{i=1}{\sum }}{\left(Y_{i}-\overset{‾}{Y}\right)}^{2}}$$

and *n* represents the number of samples, *X_i_* and *Y_i_* indicate the observed values for the two variables, and $\overset{‾}{X}$ and $\overset{‾}{Y}$ are the mean values for the two variables, respectively.

### Fusion gene validation

Total RNAs were extracted from patient BM biopsy samples of PMF, DBA, and ITP with TRIzol Reagent, and reversely transcribed using RevertAidTM First Strand cDNA Synthesis Kit (Thermo Scientific, Waltham, MA). cDNA was utilized as template to detect gene fusion with PCR amplification in Applied Biosystems using the primer pairs for *IGLV2-14-IGLL5* fusion gene: forward: 5′-AACAGCACCCAGGCAAAG-3′; reverse: 5′-TCTCCACTCCCGCCTTG-3′. The PCR products were visualized by 1.2% agarose DNA gel electrophoresis and sequenced by Beijing Genomics Institute, CAS (Beijing, China).

## Authors’ contributions

XC, XF, and ZZ conceived and designed the experiments; ND analyzed the data; ZZ, LR, WY, YZ, JZ, ZL, and PZ performed the experiments and suggested analyses; ND and ZZ wrote the paper; XZ, XC, and XF revised the manuscript. All authors read and approved the final manuscript.

## Competing interests

The authors declared that they have no conflicts of interest.

## Figures and Tables

**Table 1:** Forty-nine genes on chr19p13 or chr19q13 showing differential expression in twin brothers with childhood PMF following treatment

**Chromosomal location**	**Gene**	**Expression level (FPKM)**			**Gene**	**Expression level (FPKM)**		
		**E1**	**E2**	**Y2**		**E1**	**E2**	**Y2**
Chr19p13	*EMR1*	4.36	7.67	32.03	*PRKACA*	17.35	33.60	37.74
	*EMR4P*	1.19	1.49	8.53	*LPPR2*	17.70	29.29	38.23
	*JAK3*	14.58	27.20	67.77	*DUS3L*	10.22	8.03	4.99
	*SBNO2*	26.63	39.43	91.30	*TUBB4B*	145.66	86.30	70.89
	*BST2*	47.48	88.57	125.07	*FAM129C*	24.45	24.14	11.58
	*ZNF254*	1.21	2.33	3.16	*RETN*	2311.82	2052.35	1074.50
	*RFX2*	2.38	2.87	6.11	*NFIX*	3.09	3.68	1.35
	*CD97*	110.26	128.59	274.62	*C19orf77*	17.45	13.92	7.36
	*VAV1*	33.29	63.52	80.39	*SPC24*	9.67	6.42	2.87
	*FLJ45445*	56.41	43.17	136.00	*PRDX2*	209.35	228.38	56.45
	*FAM125A*	4.02	7.59	9.59	*KLF1*	27.88	43.17	6.02
	*PLIN5*	6.23	4.66	13.59				

Chr19q13	*FFAR3*	1.05	0.81	6.87	*CHMP2A*	92.85	165.22	207.17
	*FFAR2*	72.74	70.19	285.00	*CCDC97*	7.85	15.67	16.48
	*FPR1*	330.85	610.35	1282.02	*ZNF274*	3.55	7.06	7.30
	*RASGRP4*	24.25	46.31	76.76	*EGLN2*	37.02	64.84	75.43
	*DHX34*	8.96	16.55	25.89	*SHKBP1*	53.12	77.82	107.71
	*LILRA5*	54.72	75.30	156.33	*GMFG*	508.96	783.09	1025.10
	*CACNG6*	0	0.06	1.57	*ZNF264*	1.25	2.32	2.51
	*NUCB1*	34.86	63.55	83.02	*C19orf48*	19.13	15.94	9.11
	*FCGRT*	67.99	135.41	159.42	*ARHGAP33*	8.69	4.38	2.83
	*VASP*	102.26	176.70	238.84	*WDR62*	3.30	2.00	1.04
	*GRAMD1A*	25.08	35.30	57.30	*BLVRB*	255.04	283.60	78.55
	*ZNF224*	3.03	6.06	6.90	*APOC1*	30.95	21.16	9.34
	*KIAA0355*	1.71	2.60	3.84	*UBE2S*	33.79	20.23	9.50

*Note*: E1 and E2 denote the elder brother before and after treatment, respectively, and Y2 denotes the younger brother after treatment. Upregulated and downregulated genes are indicated in red and green, respectively. PMF, primary myelofibrosis; FPKM, fragments per kilobase of exon per million (FPKM).

**Table 2:** Gene fusions detected in the monozygotic twin brothers with childhood PMF

**Sample**	**Left**			**Right**			**No. of spanning reads**	**No. of spanning mate pairs**
	**Gene**	**Chr**	**Break-point**	**Gene**	**Chr**	**Break-point**		
Y2	*IGLV2-14*	Chr22	23101695	*IGLL5*	Chr22	23235960	19	42
E1	*IGLV2-14*	Chr22	23101695	*IGLL5*	Chr22	23235960	10	32
E2	*C22orf32*	Chr22	42475957	*FAM109B*	Chr22	42473283	8	6
Y2	*IGLV1-44*	Chr22	22735711	*IGLL5*	Chr22	23235959	7	23
Y2	*IGLV1-36*	Chr22	22786798	*IGLL5*	Chr22	23235959	7	2
E2	*IGLV2-14*	Chr22	23101695	*IGLL5*	Chr22	23235960	7	34
Y2	*C22orf32*	Chr22	42475957	*FAM109B*	Chr22	42473283	6	10
Y2	*PFKFB3*	Chr22	6268327	*LOC399715*	Chr10	6368508	6	4
Y2	*SUDS3*	Chr22	118825899	*SUDS3P1*	Chr5	177398633	5	16
Y2	*C22orf32*	Chr22	42475908	*FAM109B*	Chr22	42473283	3	10
E1	*C22orf32*	Chr22	42475906	*FAM109B*	Chr22	42473281	2	5
E1	*ELL*	Chr19	18632730	*FKBP8*	Chr19	18652804	2	5
Y2	*PARP10*	Chr8	145064223	*GRINA*	Chr8	145065395	2	3
Y2	*AC090587.5*	Chr11	3875965	*STIM1*	Chr11	3988781	2	25
E1	*AC090587.5*	Chr11	3875965	*STIM1*	Chr11	3988781	2	11
E1	*NADK*	Chr1	1711343	*SSU72*	Chr1	1500295	2	3
E1	*C22orf32*	Chr22	42476117	*FAM109B*	Chr22	42473283	1	5
Y2	*MFAP3*	Chr5	153569748	*GALNT10*	Chr5	153674375	1	6
Y2	*PRR4*	Chr12	11199784	*LOC100129361*	Chr12	11323863	1	5
Y2	*LINC00854*	Chr17	41371857	*TMEM106A*	Chr17	41371241	1	4
E2	*JAZF1*	Chr7	28031599	*JAZF1-AS1*	Chr7	28220548	1	5
E2	*LOC96610*	Chr22	22677321	*IGLL5*	Chr22	23235960	1	17
Y2	*MAP3K3*	Chr17	61767860	*LIMD2*	Chr17	61776667	1	8

*Note*: E1 and E2 denote the elder brother before and after treatment, respectively, and Y2 denotes the younger brother after treatment. PMF, primary myelofibrosis; Chr, chromosome.

## Supplementary material

Supplementary Table S1 Forty-nine genes on chr19p13 or chr19q13 showing differential expression in twin brothers with childhood PMF following treatment

Supplementary Table S2 A list of 824 genes that were differentially expressed in Y2 but expression of which remained unchanged in E2

Supplementary Table S3 SNPs in three samples annotated by SeattleSeq
