# Supplementary material for: Transcriptome Analysis of Monozygotic Twin Brothers with Childhood Primary Myelofibrosis
Source: Genomics Proteomics Bioinformatics. 2017 Feb 7;15(1):37–48. doi: 10.1016/j.gpb.2016.12.002 (PMC5339410; doi:10.1016/j.gpb.2016.12.002)
Supplement: Supplementary Table S1 — Forty-nine genes on chr19p13 or chr19q13 showing differential expression in twin brothers with childhood PMF following treatment [file mmc1.docx]

**Table S1 Clinical profiles of the monozygotic twin brother patients before, during, and after treatment**

| **Test** | **Before treatment** | | **Drug treatment** | | **After treatment** | |
| --- | --- | --- | --- | --- | --- | --- |
|  | **Y** | **E** | **Y** | **E** | **Y** | **E** |
| Systematic examination | Recurrent epistaxis, skin ecchymosis (since 1-year old), expanded abdomen (since 2-year old) | | Enlargement of liver and spleen decreased significantly | Liver and spleen enlarged increasingly, showing hepatosplenomegaly by measuring 2 cm and 15 cm below costal margins | Enlarged liver and spleen decreased to normal size | Hepatosplenomegaly |
|  | Hepatosplenomegaly by measuring 1.5 cm and 5.0 cm below costal margins (since 2-year old) | Hepatosplenomegaly by measuring 1.5 cm and 3.0 cm below costal margins (since 2-year old) |  |  |  |  |
| Complete blood counts | WBC: 6.17 × 10^9^; NEUT: 47%;  HGB: 49 g/L;  PLT: 16 × 10^9^ | WBC: 5.37 × 10^9^;  NEUT: 45%;  HGB: 49 g/L;  PLT: 23 × 10^9^ | WBC: 6.17 × 10^9^; NEUT: 3.77 × 10^9^; HGB: 141 g/L;  PLT: 40 × 10^9^;  Ret: 1.56% | WBC: 4.04 × 10^9^; NEUT: 2.73 × 10^9^; HGB: 78 g/L;  PLT: 26 × 10^9^;  Ret: 3.26% | WBC: normal; HGB: normal;  PLT: 70–80 × 10^9^ | WBC: 4.78 × 10^9^; NEUT: 2.55 × 10^9^; HGB: 72 g/L;  PLT: 45 × 10^9^;  Ret: 1.27% |
| BM smear examination | Active hyperplasia of granulocytes and RBCs; megakaryophthisis | | No difference | Active hyperplasia of granulocytes and RBCs; megakaryophthisis | Normal | Active hyperplasia of granulocytes and RBCs; megakaryophthisis |
| BM biopsy pathology | Extensive fibrous tissue hyperplasia; hyperactive hyperplasia with dominated mature granulocytes and RBCs and slightly increased immature cells | | Atypical morphology of some collagen fibrosis was less than before | Atypical morphology of some collagen fibrosis was still easily seen | Normal | Atypical morphology of some collagen fibrosis was less than before |
| Gene mutation detection | No mutation observed in *MPL*, *JAK2*-V617F, and *CARL* | | | | | |
| Karyotype | Normal | | | | | |
| Blood biochemistry | Normal | | | | | |
| Bleeding/coagulation | Normal | | | | | |
| Hemoglobin electrophoresis | Normal | | | | | |
| Coombs test | Negative | | | | | |
| G-6-PD | Normal | | | | | |
| CD55/CD59 | Normal | | | | | |
| FISH for 5q-, -5, -7, 7q-, +8, 20q- | Negative | | | | | |

*Note*: Y and E indicate the younger brother and elder brother, respectively. WBC, white blood cell; NEUT, neutrophil; HGB, hemoglobin; PLT, blood platelet; Ret, reticulocyte; BM, bone marrow; G-6-PD, glucose-6-phosphate dehydrogenase; FISH, fluorescence *in situ* hybridization; RBC, red blood cell.
